# Refining the Neonatal Phenotypic Spectrum of Distal Deletion 14q Syndrome: Early Genomic Diagnosis in Infancy

**DOI:** 10.1002/cga.70055

**Published:** 2026-04-15

**Authors:** Koji Nakae, Shiori Hamada, Junpei Kawamura, Kentaro Ueno, Toshiki Takenouchi, Yasuhiro Okamoto

**Affiliations:** ^1^ Department of Pediatrics Kagoshima University Hospital Kagoshima Japan; ^2^ Department of Pediatric Neurology Graduate School of Medicine, Dentistry and Pharmaceutical Sciences, Okayama University Okayama Japan

**Keywords:** chromosomal microarray, distal 14q deletion, exome sequencing, neonatal diagnosis, phenotypic spectrum

## Abstract

Distal deletion 14q syndrome is a rare chromosomal disorder characterized by variable features, including growth restriction, craniofacial dysmorphism, developmental delay, and congenital anomalies. Diagnosis is often delayed because conventional G‐banding may appear normal. Neonatal recognition is rarely reported, and early phenotypic features remain insufficiently defined. We report the case of a male infant born at 37 + 6 weeks of gestation with intrauterine growth restriction, micrognathia, feeding difficulties, hypotonia, and cardiopulmonary instability. Prenatal echocardiography suggested coarctation of the aorta, while postnatal imaging revealed mild bilateral pulmonary artery branch narrowing and distal aortic arch tapering without hemodynamic significance. Research‐based trio exome sequencing suggested an approximately 6.5 Mb terminal deletion of chromosome 14q32.2–q32.33, which was subsequently confirmed by chromosomal microarray. Although craniofacial features appeared subtle at birth, a retrospective review following genetic confirmation revealed additional dysmorphic traits. This case underscores the diagnostic utility of genomic testing, which enabled confirmation of distal 14q deletion at 1 month of age. In contrast, previously reported cases were typically diagnosed later in childhood. Our findings refine the neonatal phenotypic spectrum of distal deletion 14q syndrome by documenting subtle but identifiable early craniofacial, vascular, feeding, and auditory features that may prompt earlier suspicion. Early confirmation provided reassurance regarding recurrence risk and allowed for timely planning of nutritional, developmental, and audiological support. This case illustrates how genomic testing can narrow a long‐standing diagnostic gap and highlights key neonatal clues that may facilitate earlier recognition of distal deletion 14q syndrome.

## 1 Introduction

Distal deletion 14q syndrome is a rare chromosomal disorder with heterogeneous clinical manifestations, including growth restriction, craniofacial dysmorphism, developmental delay, and multiple congenital anomalies [1, 2]. The true incidence remains unknown, as available information is primarily derived from isolated case reports and small series. Although the overall phenotypic spectrum is increasingly recognized, early‐life features—particularly those present during the neonatal period—remain poorly defined. Previous reports have described a range of associated findings, including congenital heart disease, hearing impairment, and other multisystem abnormalities, underscoring the heterogeneity of the disorder [3, 4, 5]. However, most descriptions originate from later childhood or adolescence, leaving neonatal manifestations insufficiently characterized. Distal 14q deletions present significant diagnostic challenges. Conventional G‐banding may appear normal, contributing to delayed or missed diagnoses. Although chromosomal microarray analysis is recommended as a first‐tier diagnostic test for individuals with developmental disabilities or congenital anomalies [6, 7], diagnosis is often not pursued until developmental delay becomes apparent. As a result, early clinical clues that might prompt genetic testing during the neonatal period remain poorly defined.

Herein, we describe a male infant in whom a distal 14q deletion was confirmed at 1 month of age through research‐based trio exome sequencing, followed by confirmation using a clinically validated chromosomal microarray. This unusually early diagnosis highlights the utility of genomic testing in overcoming the limitations imposed by subtle neonatal features. By delineating subtle but informative neonatal findings, this case contributes to refining the early phenotypic spectrum of distal 14q deletion syndrome and illustrates how diagnosis can be achieved during infancy.

## 2 Case Report

**FIGURE 1:**
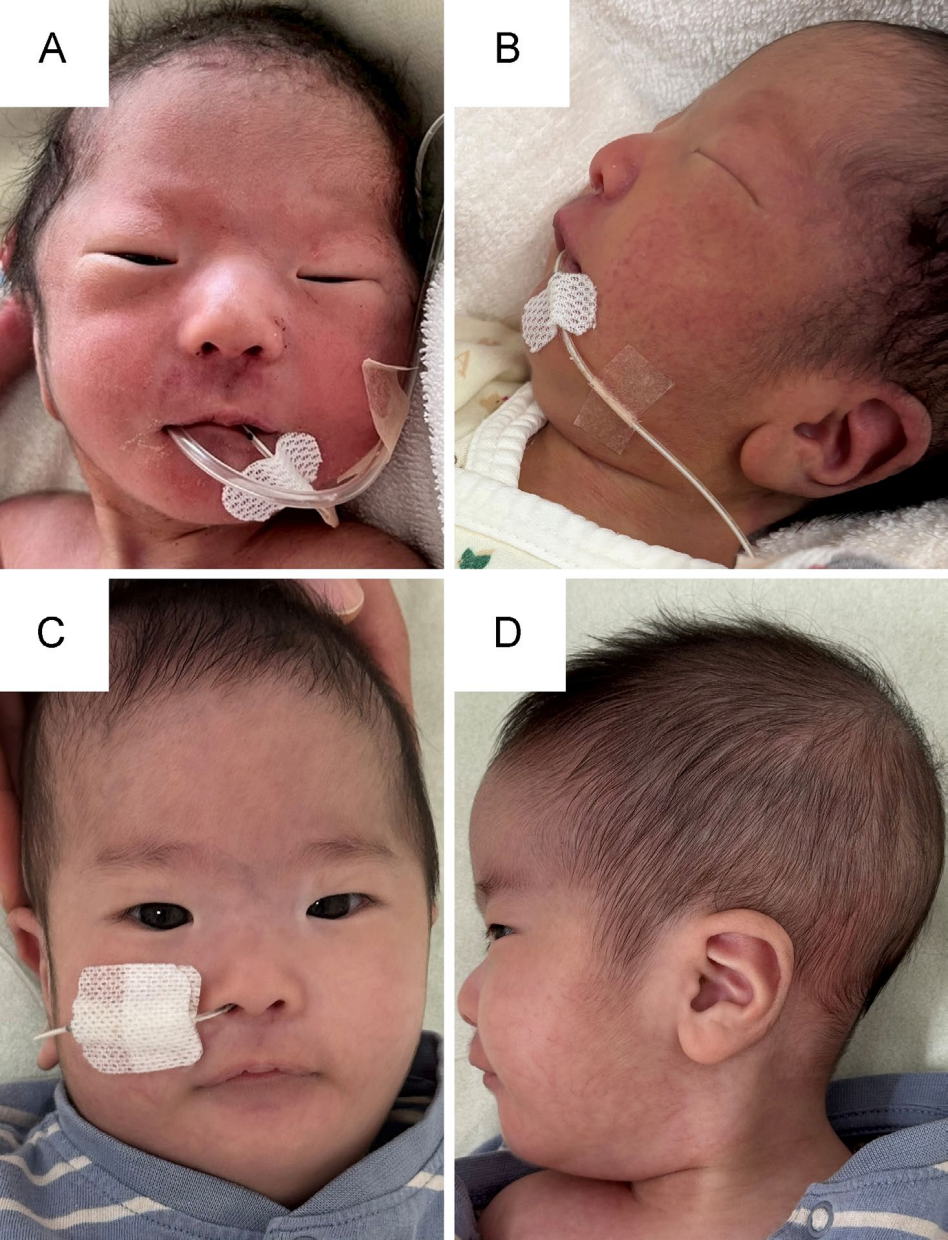
Craniofacial features at birth and at 3 months. (A) Birth, frontal view: The only clinically recognized finding at birth was micrognathia. (B) Birth, lateral view: Micrognathia and a flat facial profile were evident. (C) Three months, frontal view: Narrow palpebral fissures, high and broad forehead, a flat nasal bridge, and a long philtrum became appreciable on retrospective review following the genetic diagnosis. (D) Three months, lateral view: A flat facial profile and micrognathia are evident.

**FIGURE 2:**
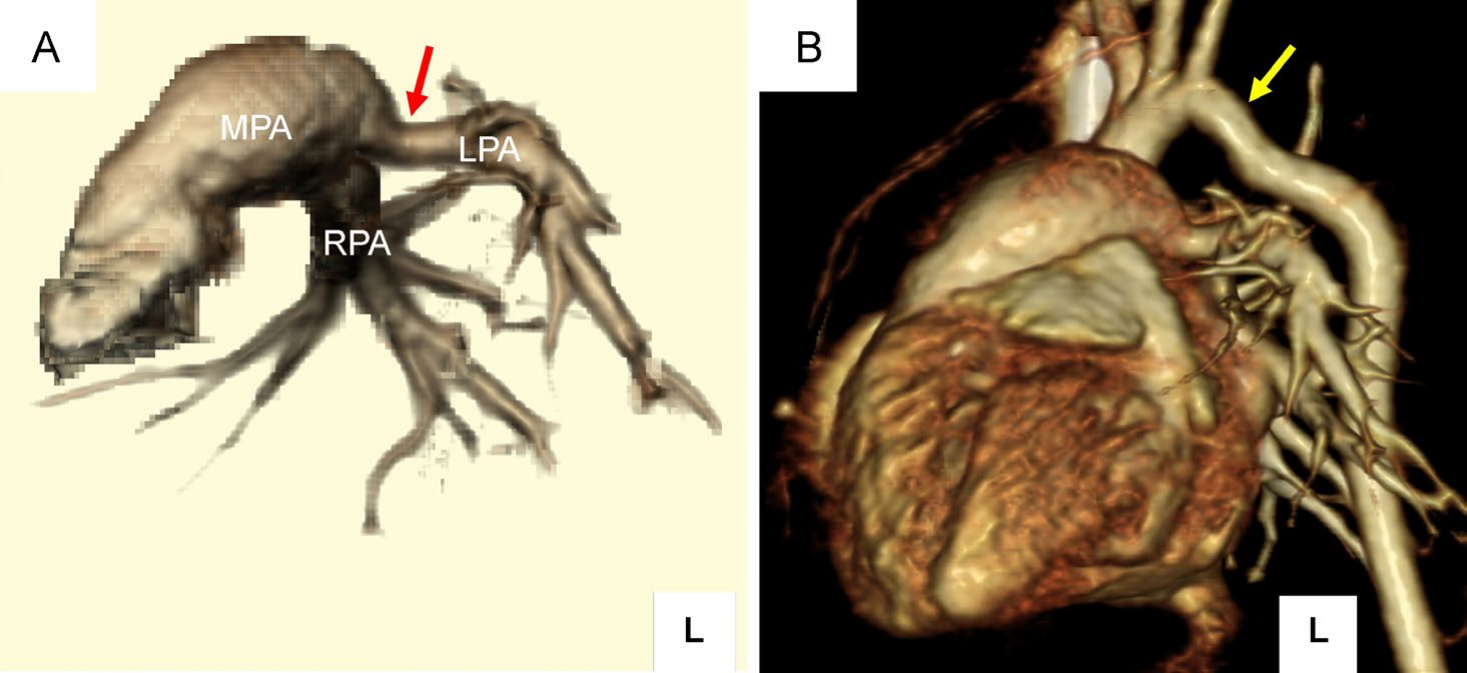
Contrast‐enhanced CT with three‐dimensional (3D) reconstructions of the great vessels. (A) 3D reconstruction of the pulmonary arteries showing mild focal narrowing of the proximal left pulmonary artery (LPA, red arrow). The main pulmonary artery (MPA) appears relatively enlarged; the right pulmonary artery (RPA) is also labeled. (B) 3D reconstruction of the thoracic aorta demonstrating mild narrowing of the distal aortic arch near the origin of the left subclavian artery (yellow arrow). Both vascular findings were mild and not hemodynamically significant.

A male infant was born at 37 weeks and 6 days of gestation via induced vaginal delivery. Prenatal ultrasonography demonstrated fetal growth restriction, without evidence of polyhydramnios. His birth weight was 2093 g (−2.2 standard deviation [SD]), length was 44.5 cm (−1.7 SD), and head circumference was 32.0 cm (−0.7 SD). Apgar scores were 6 and 8 at 1 and 5 min, respectively. Immediately after birth, the infant exhibited weak crying, hypotonia, cardiopulmonary instability, and poor oral feeding, necessitating admission to the neonatal intensive care unit (NICU) for further management. Micrognathia was observed on physical examination. The anterior fontanel was soft and normal in size, and other craniofacial features were not immediately apparent. A retrospective review following genetic diagnosis revealed additional dysmorphic features, including narrow palpebral fissures, a high and broad forehead, flat nasal bridge, flat facial profile, and long philtrum with abnormal lip morphology (Figure 1A,B). Laboratory evaluation showed polycythemia (Hb 23.9 g/dL, Ht 73.4%), requiring partial exchange transfusion, after which the clinical condition improved. Feeding remained notably poor, necessitating gavage supplementation, which continued throughout early infancy. Echocardiography revealed a mildly small left ventricle, and right ventricular pressure remained elevated without the expected postnatal decline, accompanied by mild narrowing of the pulmonary arteries. Prenatal echocardiography had suggested a possible coarctation of the aorta. Postnatal echocardiography showed a slightly narrow aortic arch; however, the findings were considered borderline. Prostaglandin E1 was not administered, and the patient was carefully monitored. After ductal closure, systemic circulation remained stable, and coarctation of the aorta was excluded. Contrast‐enhanced computed tomography (CT) on Day 24 confirmed bilateral pulmonary artery branch narrowing and mild distal aortic arch tapering without evidence of significant coarctation (Figure 2). Cardiac catheterization at 1 month of age showed a right ventricular pressure of 34/7 mmHg, main pulmonary artery pressure of 34/12 mmHg (mean, 23 mmHg), and left pulmonary artery pressure of 20/11 mmHg (mean, 15 mmHg), confirming the absence of significant pressure gradients.

The infant was discharged at 28 days of age, weighing 2356 g, and continued to require oral feeding support. An automated auditory brainstem response performed during the NICU stay showed bilateral referral, prompting otolaryngological evaluation. Diagnostic auditory brainstem response testing at 1 month confirmed bilateral hearing loss. At 3 months of age, his length was 57.5 cm (−1.7 SD), and his weight was 4120 g (−2.9 SD). The patient continued to require partial oral gavage at that time. Craniofacial features at 3 months are shown in Figure 1C,D.

**FIGURE 3:**
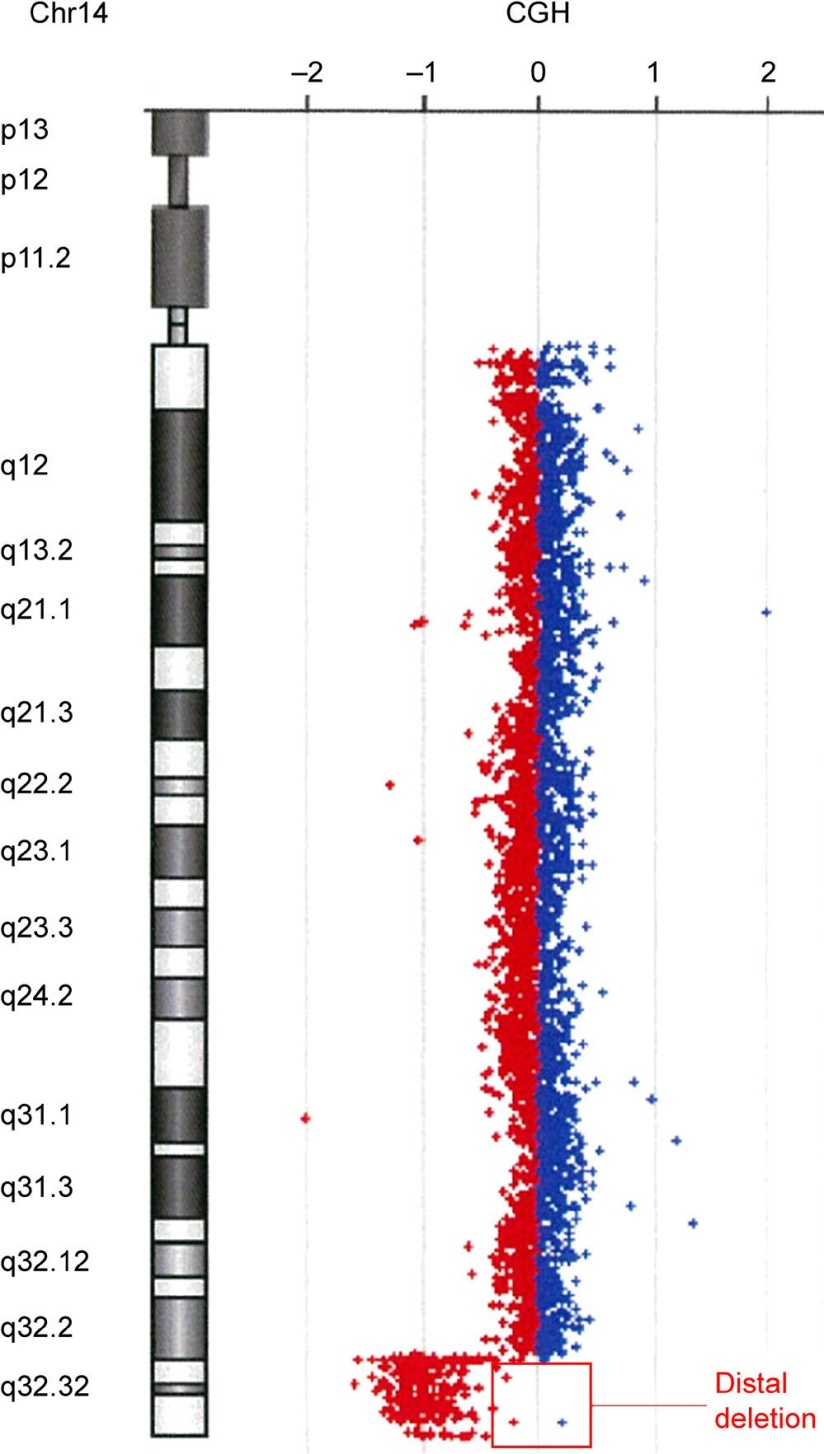
Chromosomal microarray demonstrating a distal deletion at distal 14q (~6.5 Mb). The ideogram of chromosome 14 is shown along with a CGH/SNP plot. Red‐only probes in the subtelomeric 14q region indicate copy‐number loss, consistent with a ~6.5 Mb distal deletion. Conventional G‐banding was reported as normal (46,XY). The deletion was confirmed by microarray after initial suspicion on research‐based trio exome sequencing.

At 1 month of age, in light of the presence of multiple congenital anomalies, genetic testing was initiated. Conventional G‐banding analysis was performed and showed a normal karyotype (46,XY). Research‐based trio exome sequencing, using Twist Exome 2.0 plus Comprehensive probes on genomic DNA from the patient and both parents, suggested an approximately 6 Mb distal deletion at 14q. None of the parents showed abnormalities consistent with de novo events. This finding was subsequently confirmed using a clinically validated chromosomal microarray, which identified an approximately 6.5 Mb deletion at 14q32.2–q32.33 (GRCh37: Chr14:100 801 148–107 287 505) (Figure 3). According to the American College of Medical Genetics and Genomics/ClinGen guidelines, this deletion is classified as pathogenic.

## 3 Discussion

Distal deletion 14q syndrome is associated with a heterogeneous but relatively consistent spectrum of clinical manifestations, including developmental delay, hypotonia, craniofacial dysmorphism, microcephaly, growth restriction, and various congenital anomalies(Table 1). This case has two principal points that merit discussion. First, subtle but multisystem neonatal findings prompted genetic testing and enabled diagnosis during infancy. Second, a distinctive diagnostic process—research‐based trio exome sequencing followed by confirmatory chromosomal microarray analysis—played a critical role in achieving early diagnosis despite normal conventional cytogenetic testing.

**TABLE 1:** Comparative clinical features, deletion regions, and diagnostic timing in terminal/distal 14q deletions.

References	Age at diagnosis	Sex	Size (Mb)	Developmental delay	Hypotonia	Dysmorphic features	Blepharophimosis	Microcephaly	Postnatal growth deficiency	Corpus callosum hypoplasia/agenesis	Congenital heart disease	Seizures	Inguinal/scrotal hernia	Ocular coloboma	Genitourinary anomalies	Hearing loss	Feeding difficulties
[8]	9y	F	3.2	+	+	+	−	+	+	−	−	−	−	−	−	−	−
[3]	N.R. (4y)	F	1–1.6	+	+	+	−	−	+	−	−	−	−	−	−	−	−
[7]	3y	M	4.8	+	+	+	+	+	+	+	+ ASD	+	−	N.R.	+	−	−
[7]	N.R. (4y)	M	7.6	+	+	+	−	−	−	+	−	−	+	N.R.	+	−	+
[4]	8m	M	3.13–3.19	+	−	+	+	+	+	−	+ VSD, PDA	−	+	−	+	+	+
[9]	N.R. (4y)	F	4.8	N.R.	N.R.	N.R.	N.R.	N.R.	N.R.	N.R.	N.R.	N.R.	N.R.	N.R.	N.R.	N.R.	N.R.
[10]	18y	F	4.16	+	+	+	+	+	+	−	−	−	−	−	−	+	−
[11]	N.R. (4y)	F	2.77	+	+	+	+	+	+	N.R.	−	−	−	N.R.	N.R.	−	−
[6]	N.R. (3y)	F	3.54–4.45	+	+	+	+	+	+	−	+ ASD	−	−	N.R.	−	−	−
[6]	N.R. (16y)	F	4.17	+	+	+	+	+	−	−	−	−	−	−	−	−	−
[6]	N.R. (1y)	F	3.29–3.31	+	+	+	−	−	−	−	−	−	−	−	+	−	−
[6]	N.R. (7y)	M	5.56–5.59	+	+	+	+	+	+	−	+ Supra PS	−	−	+	+	−	−
[6]	N.R. (9y)	M	5.82–5.85	+	+	+	+	−	−	−	−	−	−	−	+	−	−
[12]	N.R. (7y)	F	5.68	+	+	+	+	+	−	N.R.	−	−	−	+	−	−	−
[13]	4y	M	4.1	+	N.R.	+	+	+	−	+	−	−	+	N.R.	N.R.	−	−
[14]	12y	F	3.543	+	N.R.	+	+	−	−	+	−	−	−	N.R.	N.R.	−	−
[15]	13y	M	4.6	+	+	+	+	+	+	+	+ ASD	−	−	−	+	+	+
[16]	34y	F	3.7	+	N.R.	+	−	+	−	−	−	−	−	N.R.	−	−	−
[16]	7y	M	3.7	+	−	+	−	+	−	N.R.	−	−	−	−	−	−	−
This case	1m	M	6.5	N.R.	+	+	+	−	+	−	+ PABS	−	−	−	−	+	+

*Note:* Data in parentheses indicate the patient's age at the time of reporting when the exact age at genetic diagnosis is not specified.

Abbreviations: ASD, atrial septal defect; N.R., not reported; PABS, pulmonary artery branch stenosis; PDA, patent ductus arteriosus; PS, pulmonary stenosis; VSD, ventricular septal defect.

Most previously reported cases of distal 14q deletion syndrome have been diagnosed later in infancy or childhood, often after developmental delay became evident (Table 1). In contrast, our patient was diagnosed at 1 month of age because the coexistence of subtle neonatal findings prompted early genetic evaluation.

These findings included intrauterine growth restriction, neonatal hypotonia, feeding difficulties, mild craniofacial dysmorphism, bilateral hearing loss, and minor vascular anomalies. Individually, each feature is nonspecific; however, their combination raised clinical suspicion and enabled early diagnosis. This case therefore refines the neonatal phenotypic spectrum of distal 14q deletion by demonstrating early multisystem involvement that may otherwise appear clinically subtle. The craniofacial findings in this patient were particularly subtle during the neonatal period. At birth, only micrognathia was noted, while other traits, including narrow palpebral fissures, a high and broad forehead, flat nasal bridge, flat profile, and long philtrum, were recognized retrospectively following genetic diagnosis (Figure 1). Similar craniofacial patterns have been reported previously [5, 6, 7], though often not until later in infancy or childhood. These observations underscore the limitations of relying solely on neonatal facial morphology for clinical suspicion and highlight the complementary role of genomic testing when dysmorphic features are subtle. Cardiovascular evaluation of our patient revealed only minor anomalies. Bilateral pulmonary artery branch narrowing and distal aortic arch tapering were noted on imaging (Figure 2). Cardiac catheterization confirmed the absence of hemodynamically significant pressure gradients, supporting the classification of the vascular findings as clinically insignificant. In contrast, previously reported cases more frequently exhibit structural heart defects, such as septal defects, valve malformations, and overt arch obstruction (Table 1). Although vascular narrowing in our patient was subclinical and did not require intervention, its presence suggests that subtle vascular anomalies may be part of the neonatal spectrum of distal 14q deletion, even in the absence of overt congenital heart disease. Functional concerns further illustrate the multisystem involvement in distal 14q deletions. Feeding difficulties were evident from birth and persisted after discharge, with the patient necessitating partial gavage supplementation at 3 months. At that time, growth parameters remained below average (weight, −2.9 SD), and oral intake was insufficient. These findings are consistent with those in previous reports describing poor feeding and growth restriction in affected individuals [16]. In this case, bilateral hearing loss was identified during routine newborn hearing screening and confirmed by diagnostic Auditory Brainstem Response testing at 1 month of age. While hearing screening is universally performed and not specific to this syndrome, documentation of early‐onset hearing loss contributes to the expanding phenotypic spectrum of distal 14q deletion and may represent an under‐recognized neonatal feature. Polycythemia was also observed in the neonatal period; however, this finding has not been established as a specific feature of distal 14q deletion syndrome and may represent a nonspecific manifestation related to growth restriction or perinatal factors.

The second important aspect of this case concerns the diagnostic process. Conventional G‐banding results were normal, reflecting the known limitations of standard cytogenetics in detecting subtelomeric deletions [17]. Trio‐based exome sequencing suggested a terminal deletion at 14q, which was subsequently confirmed by chromosomal microarray analysis. Although exome sequencing is not primarily designed for copy number analysis, trio‐based evaluation of the proband and both parents provided sufficient evidence to prompt confirmatory testing. This sequential genomic approach enabled diagnosis during early infancy despite subtle phenotypic presentation; however, it was not a predefined diagnostic pathway, and further experience is needed to assess whether such an approach can be generalized [18, 19].

**FIGURE 4:**
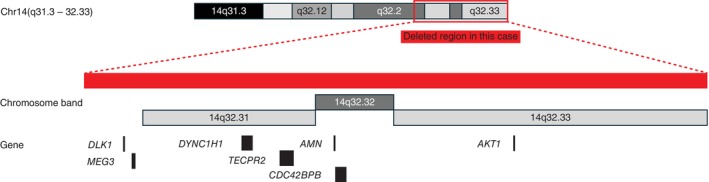
Diagram of the patient's 14q32.2–q32.33 deletion and associated genes. Schematic representation of the deleted region (14q32.2–q32.33, ~6.5 Mb). Red bars indicate the deleted intervals. Representative genes located within this region (*DLK1*, *MEG3*, *DYNC1H1*, *TECPR2*, *CDC42BPB*, *AMN*, and *AKT1*) are also shown. Although these loci have been implicated in growth regulation, cytoskeletal dynamics, autophagy, or metabolic pathways, ClinGen dosage sensitivity curation currently designates all of them as having “no evidence available” for haploinsufficiency or triplosensitivity.

The identification of a de novo deletion also has implications for family counseling. The absence of the deletion in both parents provides reassurance that the recurrence risk is low. Nevertheless, genetic counseling remains essential to address prognosis, developmental expectations, and available support resources, and to facilitate anticipatory planning for multidisciplinary care. The deletion interval in our patient (14q32.2–q32.33, GRCh37: Chr14:100 801 148–107 287 505) is schematically illustrated in Figure 4, and a more detailed genomic view is shown in Figure S1. This ~6.5 Mb region encompasses several notable genes, including the imprinted *DLK1/MEG3* domain, *CDC42BPB*, *DYNC1H1*, *TECPR2*, *AKT1*, and *AMN*. According to ClinGen curation, all of these loci are currently classified as having “no evidence available” for haploinsufficiency or triplosensitivity. Although some of these genes have been implicated in growth regulation, neurodevelopment, autophagy, cell signaling, or vitamin B12 absorption, none have been definitively linked to the full spectrum of anomalies observed in distal 14q deletions. Accordingly, the bilateral hearing loss and minor cardiovascular anomalies observed in our patient cannot be attributed to a single known gene.

In summary, the principal novelty of this case lies in the early genetic confirmation of distal 14q deletion syndrome during infancy. By shifting diagnostic attention from overt developmental delay to subtle multisystem neonatal findings, this report refines the early phenotypic spectrum of distal 14q deletion and demonstrates how genomic testing can overcome the diagnostic limitations imposed by subtle neonatal presentation.

## 4 Conclusion

This case demonstrates that distal 14q deletion syndrome can be diagnosed during infancy through genomic testing prompted by subtle multisystem neonatal findings. Early genetic confirmation refines the neonatal phenotypic spectrum of distal 14q deletion and underscores the value of considering genomic evaluation even when neonatal features are mild and nonspecific.

## Funding

This work was supported by the Japan Agency for Medical Research and Development (JP25gn0110083).

## Ethics Statement

The need for ethical approval was waived by the Institutional Review Board because this is a report of a case.

## Consent

Written informed consent for genetic testing and the publication of clinical details and photographs was obtained from the patients' parents.

## Conflicts of Interest

The authors declare no conflicts of interest.

## Supporting information


**Figure S1:** A genomic map of the deleted interval (14q32.2–q32.33; GRCh37: Chr14:100 801 148–107 287 505) was generated using the UCSC Genome Browser.

## Data Availability

Data sharing is not applicable to this article as no datasets were generated or analyzed during the current study.

## References

[1] S. J. Hreidarsson and J. Stamberg , “Distal Monosomy 14 Not Associated With Ring Formation,” Journal of Medical Genetics 20, no. 2 (1983): 147–149, 10.1136/jmg.20.2.147. 6842552 PMC1049023

[2] A. P. Ortigas , C. K. Stein , L. L. Thomson , and J. J. Hoo , “Delineation of 14q32.3 Deletion Syndrome,” Journal of Medical Genetics 34, no. 6 (1997): 515–517. 9192277 10.1136/jmg.34.6.515 PMC1050980

[3] M. L. Maurin , S. Brisset , M. Le Lorc'h , et al., “Terminal 14q32.33 Deletion: Genotype–Phenotype Correlation,” American Journal of Medical Genetics. Part A 140, no. 21 (2006): 2324–2329, 10.1002/ajmg.a.31438. 17022077

[4] K. Schlade‐Bartusiak , H. Ardinger , and D. W. Cox , “A Child With Terminal 14q Deletion Syndrome: Consideration of Genotype–Phenotype Correlations,” American Journal of Medical Genetics. Part A 149A (2009): 1012–1018. 19365838 10.1002/ajmg.a.32752

[5] C. D. M. van Karnebeek , S. Quik , S. Sluijter , M. M. F. Hulsbeek , J. M. N. Hoovers , and R. C. M. Hennekam , “Further Delineation of the Chromosome 14q Terminal Deletion Syndrome,” American Journal of Medical Genetics 110, no. 1 (2002): 65–72, 10.1002/ajmg.10207. 12116274

[6] H. Engels , H. M. Schüler , A. M. Zink , et al., “A Phenotype Map for 14q32.3 Terminal Deletions,” American Journal of Medical Genetics. Part A 158A, no. 4 (2012): 695–706, 10.1002/ajmg.a.35256. 22367666

[7] A. Schneider , B. Benzacken , A. Guichet , et al., “Molecular Cytogenetic Characterization of Terminal 14q32 Deletions in Two Children With an Abnormal Phenotype and Corpus Callosum Hypoplasia,” European Journal of Human Genetics 16, no. 6 (2008): 680–687, 10.1038/sj.ejhg.5201977. 18197200

[8] K. Schlade‐Bartusiak , T. Costa , A. M. Summers , M. J. Nowaczyk , and D. W. Cox , “FISH‐Mapping of Telomeric 14q32 Deletions: Search for the Cause of Seizures,” American Journal of Medical Genetics. Part A 138A, no. 3 (2005): 218–224, 10.1002/ajmg.a.30942. 16152642

[9] M. Zollino , L. Seminara , D. Orteschi , et al., “The Ring 14 Syndrome: Clinical and Molecular Definition,” American Journal of Medical Genetics. Part A 149A, no. 6 (2009): 1116–1124, 10.1002/ajmg.a.32831. 19441122

[10] E. L. Youngs , J. A. Hellings , and M. G. Butler , “A Clinical Report and Further Delineation of the 14q32 Deletion Syndrome,” Clinical Dysmorphology 20, no. 3 (2011): 143–147, 10.1097/MCD.0b013e3283438200. 21358539 PMC7360343

[11] D. C. Darcy , S. Rosenthal , and R. J. Wallerstein , “Chromosome Deletion of 14q32.33 Detected by Array Comparative Genomic Hybridization in a Patient With Features of Dubowitz Syndrome,” Case Reports in Genetics 2011 (2011): 306072, 10.1155/2011/306072. 23074674 PMC3447229

[12] A. Jezela‐Stanek , M. Kucharczyk , A. Gutkowska , et al., “History and Molecular Characteristics of a Patient With Terminal Deletion of 14q: Is This Another Syndrome With a Striking Phenotype?,” Clinical Dysmorphology 21, no. 2 (2012): 97–100, 10.1097/MCD.0b013e32834e92b8. 22391620

[13] G. Bağcı , G. O. Çetin , N. Semerci , G. A. Toruner , and M. Cinbiş , “Terminal 14q Deletion With Unbalanced t(Y;14)(q12;q32) Translocation,” Clinical Dysmorphology 21, no. 1 (2012): 37–41, 10.1097/MCD.0b013e32834d6ba3. 22143350

[14] B. A. Mendelsohn , L. L. B. Jeng , S. Oberoi , and O. D. Klein , “Dental Findings in 14q Terminal Deletion Syndrome,” Clinical Dysmorphology 23, no. 2 (2014): 60–62, 10.1097/MCD.0000000000000026. 24535103

[15] M. E. Amodeo , E. Inzaghi , A. Deodati , and S. Cianfarani , “Endocrinological Features of a Patient With 14q Microdeletion and Dubowitz Phenotype,” Molecular Genetics & Genomic Medicine 9, no. 5 (2021): e1644, 10.1002/mgg3.1644. 33788412 PMC8172207

[16] K. M. Vincent , B. Prince , J. McGowan‐Jordan , and M. T. Carter , “Familial Inheritance of 14q Terminal Deletion Syndrome and Review of the Literature,” European Journal of Medical Genetics 75 (2025): 105015. 40180153 10.1016/j.ejmg.2025.105015

[17] D. T. Miller , M. P. Adam , S. Aradhya , et al., “Consensus Statement: Chromosomal Microarray Is a First‐Tier Clinical Diagnostic Test for Individuals With Developmental Disabilities or Congenital Anomalies,” American Journal of Human Genetics 86, no. 5 (2010): 749–764, 10.1016/j.ajhg.2010.04.006. 20466091 PMC2869000

[18] K. Retterer , J. Juusola , M. T. Cho , et al., “Clinical Application of Whole‐Exome Sequencing Across Clinical Indications,” Genetics in Medicine 18, no. 7 (2016): 696–704, 10.1038/gim.2015.148. 26633542

[19] E. R. Riggs , E. F. Andersen , A. M. Cherry , et al., “Technical Standards for the Interpretation and Reporting of Constitutional Copy‐Number Variants: A Joint Consensus Recommendation of the American College of Medical Genetics and Genomics (ACMG) and the Clinical Genome Resource (ClinGen),” Genetics in Medicine 22, no. 2 (2020): 245–257, 10.1038/s41436-019-0686-8. 31690835 PMC7313390

