# Supplementary figures and images for: Refining the Neonatal Phenotypic Spectrum of Distal Deletion 14q Syndrome: Early Genomic Diagnosis in Infancy
Source: Congenit Anom (Kyoto). 2026 Apr 15;66(1):e70055. doi: 10.1002/cga.70055 (PMC13092366; doi:10.1002/cga.70055)

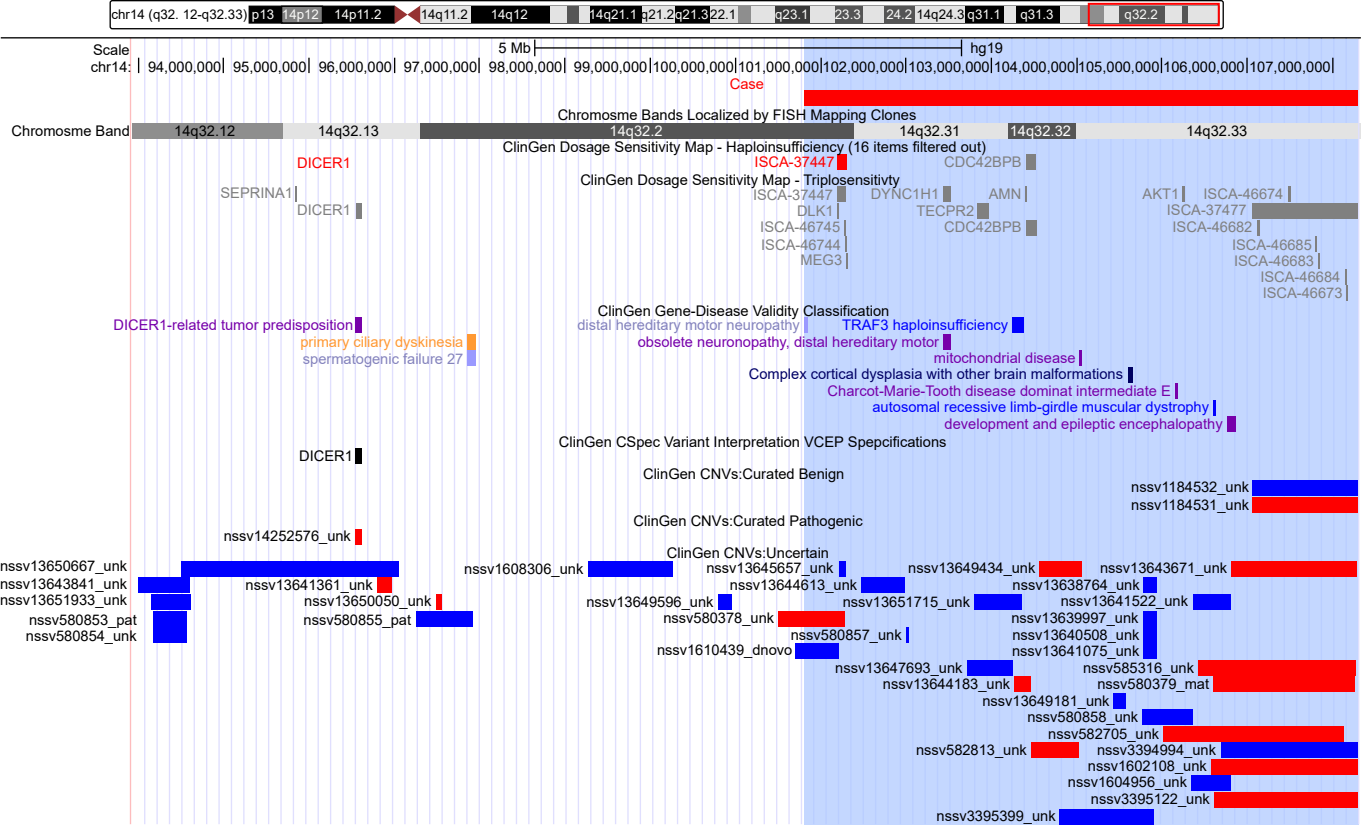

Supplement: Supplementary file 1 — Figure S1: A genomic map of the deleted interval (14q32.2–q32.33; GRCh37: Chr14:100 801 148–107 287 505) was generated using the UCSC Genome Browser. [file CGA-66-0-s001.pdf]
